# Simultaneous Analysis of Biomarkers in Human Hair for Evaluating Chronic Tobacco Smoke Exposure and Stress/Relaxation Using Online In-Tube Solid-Phase Microextraction Coupled with Liquid Chromatography–Tandem Mass Spectrometry

**DOI:** 10.3390/molecules31050770

**Published:** 2026-02-25

**Authors:** Hiroyuki Kataoka, Akiko Tsuzaki, Sae Kitagawa, Kentaro Ehara

**Affiliations:** School of Pharmacy, Shujitsu University, Okayama 703-8516, Japan

**Keywords:** tobacco smoke exposure, stress, biomarkers, hair, in-tube solid-phase microextraction (IT-SPME), liquid chromatography–tandem mass spectrometry (LC–MS/MS)

## Abstract

Tobacco smoke exposure not only increases the risks of lung cancer and cardiovascular disease, but can be a stressor contributing to mental illness. It is important to clarify the relationship between chronic tobacco smoke exposure and mental stress from the perspective of disease prevention. We developed a simple and highly sensitive method for simultaneously analyzing nine biomarkers: nicotine and cotinine (tobacco smoke exposure markers); cortisol, testosterone, and dehydroepiandrosterone (stress-related markers); and serotonin, melatonin, dopamine, and oxytocin (relaxation-related markers). Biomarkers were extracted and concentrated by in-tube solid-phase microextraction with a Supel-Q PLOT capillary, followed by separation and detection within 7 min using liquid chromatography–tandem mass spectrometry on a Discovery HS F5 column. Calibration curves using stable isotope-labeled internal standards showed good linearity (0.005–100 ng mL^−1^) with detection limits of 0.09–13.5 pg mL^−1^. Intra-day and inter-day precision had relative standard deviations below 7.2% and 15.5% (*n* = 6), respectively, with recovery rates of 84.0–108.8%. The automated method requires only ultrafiltration of hair methanol extract, enabling non-invasive pg-level analysis using just a few milligrams of hair. Hair analysis reflects an association between chronic tobacco smoke exposure and stress. This method is effective for analyzing the relationship between long-term tobacco smoke exposure and chronic stress.

## 1. Introduction

In addition to active smoking, the risk factors of passive smoking from side stream tobacco smoke and exhaled smoke, as well as thirdhand smoking from environmental tobacco smoke residue on clothing, curtains, and wallpaper, are significant risk factors for lung cancer, cardiovascular disease, and respiratory diseases. These risk factors represent serious health and public health concerns [1,2,3,4,5,6,7,8]. Furthermore, exposure to tobacco smoke not only increases the risk of tobacco-related diseases, but it is also associated with various stress-related mental disorders, such as anxiety and depression [9,10,11,12,13]. Smokers often believe that the nicotine (Nic) in tobacco smoke acts on the central nervous system to release dopamine (DA), which relaxes tension and provides pleasurable feelings. However, the actual half-life of Nic in the blood is short (approximately 30 min), and as Nic levels drop, withdrawal symptoms (stress-like symptoms such as irritability and aggression) appear. Smokers are therefore thought to become dependent on Nic to escape this withdrawal [11,12,13]. Thus, the stress-relieving effect of smoking is temporary, and quitting smoking has been reported to actually reduce stress [14]. In contrast, for non-smokers, the unpleasant odor and irritation of tobacco smoke may act as stressors, and individuals exposed through passive smoking reportedly experience more irritability and depression than non-exposed individuals [15]. While the experience of smoking or passive smoking may be subjectively perceived as either stressful or relaxing depending on the individual, objective quantification of these physiological states remains limited. Since stress can trigger neuropsychiatric disorders, cardiovascular disease, gastric ulcers, and depression [16,17,18,19,20,21], objectively quantifying the degree of stress or relaxation induced by active or passive smoking is important for assessing the mental health effects of tobacco smoke exposure.

Approximately 70–80% of Nic absorbed from tobacco smoke is metabolized into cotinine (Cot). Both Nic and Cot are detectable in body fluids (blood, saliva, urine) and hair, making them widely used as specific biomarkers for tobacco smoke exposure [2,7]. Cor-tisol (CRT), the end product of the hypothalamic–pituitary–adrenal (HPA) axis, is a key stress response hormone widely used as a biomarker of stress [16,17]. Numerous previous studies have suggested an association between smoking and the HPA system, which is implicated in both stress response and Nic dependence [7,22]. Nic is thought to induce CRT production [22,23,24]. Furthermore, HPA axis responses may be influenced by stress-related steroid hormones such as testosterone (TES) and dehydroepiandrosterone (DHEA) [25,26,27]. Various endocrine hormones and autonomic nervous system neurotransmitters have been investigated as stress biomarkers [16,17,28,29,30,31,32,33,34]. However, since psychological stress fluctuates with pleasant or unpleasant emotions and manifests acutely or chronically, biomarkers associated with relaxation such as DA (pleasure and elation), serotonin (5-HT; security and happiness), melatonin (MEL; sleep induction), and oxytocin (OXT; warmth and affection) could also serve to estimate relaxed states [29,30,33,35,36,37,38]. Decreased 5-HT indicates anxiety tendencies, MEL fluctuations suggest sleep disorders, elevated DA reflects reward system activity, and increased OXT signifies relaxation. Combining these with CRT levels may provide comprehensive assessment of stress and relaxation states. However, since concentrations of these biomarkers in biological samples are extremely low, highly sensitive analytical instruments and efficient sample pretreatment methods are essential for reliable measurements. Furthermore, analyzing the relationship between tobacco smoke exposure and stress requires simultaneous analysis of biomarkers that specifically respond to each.

Recently, we developed a simple and sensitive liquid chromatography–tandem mass spectrometry (LC–MS/MS) method for simultaneously measuring the tobacco smoke exposure markers Nic and Cot, the stress biomarker CRT, and various relaxation-related biomarkers, and applied it to saliva sample analysis [39]. This method enables online automated analysis by incorporating in-tube solid-phase microextraction (IT-SPME), an efficient sample concentration technique, into the system. Using this method, we demonstrated a positive correlation between salivary Nic concentration and CRT concentration in non-smokers. Additionally, we found a negative correlation between SDNN (standard deviation of normal-to-normal intervals) values, derived from heart rate variability analysis, and CRT concentration. These results suggest that tobacco smoke exposure acts as a stressor, consistent with previous findings [23,25]. However, although saliva samples can be collected non-invasively, biomarker half-lives are short due to metabolism and excretion [25,40,41]. Saliva samples are also easily affected by diet, and biomarker concentrations fluctuate greatly due to circadian rhythms [23,37,42]. Therefore, while saliva samples can evaluate the effects of short-term tobacco smoke exposure, they are not suitable for assessing the effects of long-term exposure, such as chronic smoking or passive smoking, on stress.

In contrast, Nic and other biomarkers are incorporated into hair shafts via diffusion from hair follicles during blood circulation and become fixed there. They remain relatively stable and are less affected by metabolism and excretion. Therefore, hair serves as an effective biological sample for evaluating long-term tobacco smoke exposure and chronic stress states [7,8,43,44,45,46,47,48,49,50,51,52,53]. Nic and Cot in hair are widely used as biomarkers for long-term tobacco smoke exposure [7,8,23,44,45], while hair CRT and related steroid hormones serve as biomarkers for chronic stress [23,41,46,47,48,49,50,51,52,53]. Since hair typically grows at 1 cm per month, biomarker concentrations per centimeter reflect approximately one month of past body levels. Therefore, segment analysis specifying length from the hair root can identify the exposure period and body levels during that time. Analyzing biomarkers across the entire hair strand allows estimation of average exposure (cumulative exposure divided by exposure period) and long-term average body concentrations [37,41,44,52,53]. Furthermore, hair samples can be collected non-invasively and painlessly, are unaffected by diurnal variations, and pose minimal biohazard concerns. Hair also offers additional advantages: its keratin coating protects against oxidation and thermal degradation, allowing stable long-term storage at room temperature in a dry, dark environment [43]. However, caution is required because hair type and color vary by race and age, and hair is susceptible to chemical and physical treatments such as harsh detergents and perms [23,53].

Numerous analytical methods have been reported for Nic and Cot [23,40,54,55,56,57,58,59,60,61,62,63,64,65,66] as well as CRT and stress-related hormones [22,24,27,37,41,42,67,68,69,70,71,72,73,74,75] in human hair. While one report [23] addresses both tobacco smoke exposure markers and CRT, it measures them separately; no method for simultaneous analysis has been found. Among these reports, representative methodologically focused papers are summarized in Table 1, with the characteristics of each method detailed. An excellent review on sample handling and pretreatment methods for hair analysis has also been published [43].

The procedure for analyzing biomarkers in hair samples requires a series of steps: sampling, washing to remove external contaminants, extraction, concentration, purification of biomarkers, and instrumental separation and detection. However, the reported methodologies vary widely, and no standardized protocols exist. Washing hair samples is necessary to remove analytes adhering to the hair surface and external contaminants such as sweat, sebum, and hair care products. For Nic and Cot analysis, dichloromethane [54,55,56,59,63,64,65] or isopropanol [23] is generally used, while for CRT and steroid hormone analysis, water [67,68], acetone [68], methanol [37,41,53,69], or isopropanol [23,66,70] is typically employed. Liquid–liquid extraction (LLE) [54,55,56,59], microwave-assisted extraction (MAE) [65], solid-phase extraction (SPE) [23,66,68], and SPME [63,64] have been used to extract biomarkers from hair samples and remove coexisting substances before analysis. For Nic and Cot, methods include NaOH digestion followed by dichloromethane extraction [54,55,56,59], MAE using methanol/trifluoroacetic acid [65], methanol extraction followed by SPE [23], and water extraction followed by IT-SPME [63,64]. However, NaOH digestion causes instability of amide bonds in nicotinamide, resulting in high chromatographic background noise, poor recovery rates, and the need for additional processing steps such as LLE or SPE [23,71]. In contrast, methanol swells the hair structure and releases biomarkers, allowing the methanol extract to be injected directly into an LC–MS system without additional pretreatment [23,71]. For CRT and steroid hormones, methods include methanol extraction [38,68], post-extraction SPE [41,53,67,72,73], and post-SPE derivatization [70]. Among these pretreatment methods, LLE and offline SPE are complex and time-consuming, while online SPE [23,67,72] and IT-SPME [39,64,65] are easily automatable by direct coupling with HPLC.

Nic and Cot in human hair samples have been measured by gas chromatography–mass spectrometry (GC–MS) [57,58,59], high-performance liquid chromatography (HPLC), LC–MS, or LC–MS/MS [8,10,20,21,23,39,60,64,65,66]. In contrast, CRT and other stress- and relaxation-related biomarkers in hair have predominantly been measured by LC–MS/MS [24,37,41,49,50,51,52,67,68,69,70,72,73,74,75,76]. Among these methods, HPLC exhibits insufficient sensitivity and selectivity, whereas GC–MS demonstrates excellent sensitivity and selectivity but requires laborious derivation to convert analytes into volatile derivatives. LC–MS and LC–MS/MS methods enable selective, sensitive, and high-throughput analysis of these biomarkers without derivatization. However, most techniques require cumbersome and time-consuming offline sample pretreatment, such as LLE or SPE, to prevent column clogging or mass spectrometer contamination. Currently, the most effective approaches combine SPE or SPME with LC–MS/MS online via column switching. However, no methods have been reported for simultaneous measurement of tobacco smoke exposure biomarkers and stress-/relaxation-related biomarkers in hair.

**Figure 1:**
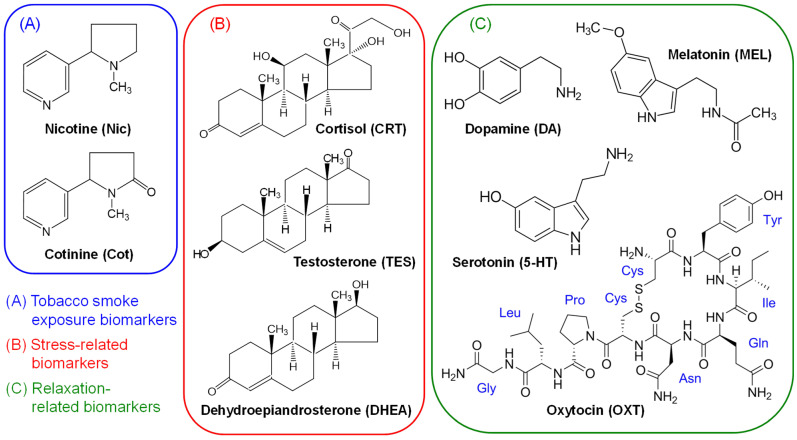
Structures of tobacco smoke exposure biomarkers and stress- and relaxation-related biomarkers.

IT-SPME is a sample preparation technique using an internally coated open tubular fused silica capillary as an extraction device. It automates sampling through extraction and concentration, facilitating online coupling to HPLC or LC–MS via column switching [77,78]. This method significantly simplifies conventional sample preparation due to its compact size, ease and speed of operation, automation capability, environmental friendliness, and low solvent consumption, making it widely applicable when extracting compounds from complex biological matrices [79]. We previously established IT-SPME analytical methods for tobacco smoke exposure biomarkers in saliva [39] and stress/relaxation biomarkers [34,39,78,80], and demonstrated their applicability to hair sample analysis [64,65,81,82]. Therefore, this study aimed to develop a highly sensitive simultaneous analysis method using IT-SPME LC–MS/MS for Nic and Cot (tobacco smoke exposure biomarkers), TES, DHEA, and CRT (stress-related biomarkers), and 5-HT, MEL, DA, and OXT (relaxation-related biomarkers), as shown in Figure 1, and to elucidate the causal relationship between long-term tobacco smoke exposure and chronic stress. The developed method was applied to hair analysis of non-smokers and smokers as a pilot test to evaluate its validity.

**Table 1:** Main analytical methods for the determination of human hair biomarkers targeted in this study.

Analyte ^1^	Sample Preparation	Detection ^2^	Sensitivity ^3^	Precision RSD (%)	Recovery (%)	Content ^4^ (ng mg^−1^)	Ref.
Nic	Hair samples (approximately 30 mg) collected from the scalp at a 3 cm distance were washed using 3 mL of CH_2_Cl_2_ and sonicated for 30 min. Dried hair samples were digested by heating in 1.5 mL of 1 M NaOH solution at 50 °C for 24 h, then extracted twice with CH_2_Cl_2_ after adding 17.5 μL of octanol. The solvent in the extract was evaporated to dryness and dissolved in 52.5 μL of MeOH.	Isotope dilution GC–MS-SIM	LOD (pg mg^−1^) Nic: 20	Within-batch: 6.3–8.5 Between-batch: 11.9–20.9	Within-batch: 84.8–88.1 Between-batch: 73.5–83.3	Non-smokers Women (40): 0.42–1.00 Children (40): 0.88–2.08	[54,55,56]
Nic, Cot	Full length of hair samples (10–100 mg) from the occipital region of the scalp were washed twice with water, hexane and CH_2_Cl_2_, and dried in an oven at 50 °C. Dried hair samples were digested by heating in 2 mL of 1 M NaOH solution at 60 °C for 90 min, then extracted twice with 3 mL of CH_2_Cl_2_ for 15 min. The solvent in the extract was evaporated to dryness by N_2_ at 30 °C after addition of 50 μL of 1 M HCl, and then dissolved in 100 μL of MeOH.	LC–MS-SIM	LOD (pg mg^−11^) Nic: 20 Cot: 50 LOQ (pg mg^−1^) Nic: 15 Cot: 50	Within-run Nic: 12.4–15.6 Cot: 16.5–19.4 Between-run Nic: 4.2–21.5 Cot: 3.3–23.3	Nic: 69.15 Cot: 72.08	Infants (22) Nic: 0.18–28.71 Cot: 0.13–1.57 Adults Non-smokers (19) Nic: 0.12–2.58 Cot: 0.05–0.32 Smokers (25) Nic: 2.01–79.30 Cot: 0.08–2.49	[60]
Nic, Cot	Full length of hair samples (5 mg) from the scalp were thrice washed with 1.mL of CH_2_Cl_2_ and dried in an oven at 50 °C. Dried hair samples (1–2 mg) were extracted with 1 mL of distilled water at 80 °C for 30 min.	Online IT-SPME LC–MS/MS-MRM	LOD (pg mL^−1^) Nic: 0.45 Cot: 0.13 LOQ (pg mg^−1^) Nic: 7.5 Cot: 4.4	Intra-day Nic: 1.62–1.64 Cot: 3.12–3.37 Inter-day Nic: 2.42–5.97 Cot: 2.35–2.49	Nic:95.0–96.1 Cot: 87.0–96.6	Non-smokers (50) Nic: 1.88 ± 1.85 Cot: 0.022 ± 0.028 Smokers (8) Nic: 43.12 ± 34.81 Cot: 0.655 ± 0.616	[64,65]
Nic, Cot	Hair samples from the scalp were washed twice with 2 mL of CH_2_Cl_2_ and cut into pieces (≤1 mm) with scissors, weighed (20 mg), placed into the glass tube, and then extracted with 0.5 mL MeOH/TFA (8.5:1.5, *v*/*v*) by microwave-assisted extraction (MAE) for 3 min. After evaporation to dryness by N_2_ at 40 °C, the dried residues were reconstituted in 100 μL MeOH.	LC–MS/MS-SRM	LOD (pg mg^−1^) Nic: 2 Cot: 4 LOQ (pg mg^−1^) Nic: 5 Cot: 10	Intra-day Nic: 4.4–6.2 Cot: 3.1–7.6 Inter-day Nic: 3.4–5.6 Cot: 2.4–6.7	Accuracy (% bias) Intra-day Nic: 4.1–7.0 Cot: −3.8–8.0 Inter-day Nic: 5.8–7.5 Cot: 4.1–8.2	Smokers (8) Nic: 3.83–33.42 Cot: 1.08–6.08 Black hair (3) Nic: 5.22–19.86 Cot: 1.81–2.33 Glay hair (3) Nic: 1.65–5.18 Cot: 0.45–0.89	[66]
Nic, Cot	Hair samples cut as 3 cm segments from the scalp at the posterior vertex were washefinee in 2.5 mL isopropanol at room temperature and dried under a fume hood for 12 h. 7.5 mg of the dried whole hair was sliced into small pieces using fine scissors, and then extracted with 1.8 mL MeOH for 18 h at room temperature. After evaporation to dryness by N_2_ at 50 °C, the dried residues were reconstituted in 225 μL 50% MeOH.	Online SPE LC–MS/MS-MRM	LOQ (pg mg^−1^) Nic: 1 Cot: 0.1	Intra-day Nic: 5.2–12.2 Cot: 3.2–8.1 Inter-day Nic: 6.6–11.4 Cot: 6.0–8.7	Nic:94–103 Cot: 90–104	Non-smokers (20) Nic: 7.54 ± 8.61 Cot: 2.99 ± 2.43 Smokers (20) Nic: 732.7 ± 1424.7 Cot: 279.3 ± 384.7	[23]
7 steroid hormones (containing CRT, TES, DHEA)	Hair samples (20 mg) cut as 3 cm segments from the scalp at the posterior vertex were washed with 2.5 mL isopropanol for 3 min at room temperature and dried under a fume hood for 12 h. 10 mg of the dried hair was extracted with 1.8 mL MeOH for 18 h at room temperature. After evaporation to dryness by N_2_ at 65 °C, the dried residues were reconstituted in 250 μL distilled water.	Column-switching online SPE LC–APCI–MS/MS	LOQ (pg mg^−1^) CRT: 0.09 TES: 0.08 DHEA: 0.9	Intra-day CRT: 3.7–8.4 TES: 2.5–6.2 DHEA: 4.5–9.1 Inter-day CRT: 3.2–5.3 TES: 3.1–8.3 DHEA: 6.3–8.5	CRT: 89–93 TES: 93–97 DHEA: 78–85	(pg mg^−1^) Men (15) CRT: 2.30–17.64 TES: 1.16–4.18 DHEA:7.36–30.0 Women (15) CRT: 1.62–13.86 TES: 0.64–2.82 DHEA:5.34–42.5	[67]
CRT	Hair samples from the posterior vertex were washed with 15 mL water for 3 min and then 10 mL acetone for 2 min, and finally dried at room temperature for 4 h. The dried hair was extracted with 5 mL MeOH for 16 h in an ultrasonic bath at 55 °C. After evaporation to dryness by N_2_ at 35 °C, the dried residues were reconstituted in 150 μL MeOH and 350 μL.	LC–MS/MS-MRM	LOD (pg mg^−1^) CRT: 0.2 LOQ (pg mg^−1^) CRT: 1.0	Intra-day:3.1–7.5 Inter-day:6.0–8.8	CRT: 92.7–102.3	(pg mg^−1^) CRT: 3.2	[68]
9 steroid hormones (containing CRT, TES)	Hair samples (10–20 mg) were washed with 1 mL water and 1 mL acetone, and the dried hair was extracted with 1.4 mL MeOH for 24 h. After evaporation to dryness, the dried residues were reconstituted in 120 μL water.	Online SPE LC–MS/MS-MRM LC–MS/MS/MS	LOD (pg mL^−1^) CRT: 10.1 TES: 4.9 LLOQ (pg mg^−1^) CRT: 36.2 TES: 6.3	Intra-day CRT: 10.5–16.9 Inter-day CRT: 15	CRT: 82	(pg mg^−1^) CRT: 4.1	[72]
CRT cortisone	Hair samples (≥1 cm) from the posterior vertex region were cut with iron scissors, and washed with 5 mL of MeOH for 2 min at room temperature. 20 mg of hair was extracted with 1 mL of MeOH at 40 °C for 24 h. After evaporation to dryness by N_2_, the dried residues were reconstituted in 50 μL MeOH and 1 mL water, and cleaned up with an SPE C18 column.	LC–APCI–MS/MS-MRM	LOQ (pg mg^−1^) CRT: 0.5 Cortisone: 1.0	Intra-day: <10% Inter-day: <10%	95–105	(pg mg^−1^) Female college students (44) CRT: 0.8–22.5 Cortisone: 8.3–59.2	[73]
8 steroid hormones (containing CRT, TES, DHEA)	Hair segments (1 cm) closest to the scalp were washed twice in 3 mL MeOH for 2 min and dried at room temperature in a fume cupboard overnight. 20 mg of hair was extracted with 1 mL of MeOH at 25 °C for 24 h. After evaporation to dryness by N_2_, the dried residues were reconstituted in 100 μL water and 1 mL MeOH, and cleaned up with an SPE C18 column.	SPE UPLC– APCI–MS/MS-MRM	LOD (pg mg^−1^) CRT: 0.3 TES: 0.4 DHEA: 0.6 LOQ (pg mg^−1^) CRT: 0.8 TES: 1.0 DHEA: 1.3	Intra-day CRT: 4.9–8.0 TES: 5.2–5.7 DHEA: 7.4–9.6 Inter-day CRT: 5.9–7.8 TES: 3.7–9.9 DHEA: 8.6–10.2	CRT: 97.3–99.3 TES: 86.9–99.4 DHEA: 85.5–107.5	(pg mg^−1^) Participants (308) CRT: 0.8–49.1 TES: 1.8–69.0 DHEA:1.9–58.3	[41,53]
22 steroid hormones (containing CRT, TES, DHEA)	Hair samples (10–15 mg) of whole hair (3 cm closest to scalp) were washed with 1.5 mL isopropanol for 2 min and the dried hair was extracted with 1.5 mL MeOH for 18–20 h at room temperature. After evaporation to dryness by N_2_ at 40 °C, the dried residues were reconstituted in 1 mL 30% MeOH. Hair extracts were subsequently cleaned by SPE using Oasis HLB. The dried SPE sample was cleaned up by LLE using NaCl solution and toluene, and then derivatized with 100 mM hydroxylamine hydrochloride at 60 °C for 30 min.	SPE/LLE and hydroxylamine derivatization UPLC–HRMS/MS	LLOQ (pg mg^−1^) CRT: 1.5 TES: 0.1 DHEA: 0.3	Intra-day CRT: 2 TES: 4 DHEA: 3 Inter-day CRT: 2 TES: 3 DHEA: 2	CRT: 96 TES: 90 DHEA: 100	(pg mg^−1^) Participants (8) CRT: 422 TES: 92 DHEA:290	[70]
5 biomarkers (containing CRT, MEL)	Hair samples from the scalp were washed twice with 2.mL MeOH and the dried hair samples were cut into small pieces (1–2 mm) with scissors. The cut hair samples (20 mg) were placed into the glass tube, and then extracted with 1 mL of MeOH at 27 °C for 24 h. After evaporation to dryness by N_2_ at 26 °C, the dried residues were reconstituted in 50 μL 95% MeOH.	LC–QTRAP MS/MS	LOD (pg mg^−1^) CRT: 0.1 MEL: 0.05 LOQ (pg mg^−1^) CRT: 0.8 MEL: 0.5	Intra-day CRT: 1.7–9.7 MEL: 1.8–11.5 Inter-day CRT: 6.6–11.5 MEL: 6.0–10.2	CRT: 101.5–111.7 MEL: 105.3–113.4	(pg mg^−1^) Participants (65) CRT: 0.0–8.8 MEL: 0.05–0.82	[37]
Nic, Cot, TES, DHEA, CRT, 5-HT, MEL, DA, OXT	Full length of hair samples (15 mg) from the scalp were washed twice with 1.mL of CH_2_Cl_2_ by sonication for 3 min, and dried at room temperature. The dried hair samples were cut into small pieces (2–3 mm) with scissors; 5 mg was extracted with 0.6 mL MeOH at 40 °C for 24 h in a screw-cap vial.	Online IT-SPME LC–MS/MS-MRM	LOD (pg mg^−1^) Nic: 0.05 Cot: 0.02 TES: 0.05 DHEA: 0.31 CRT: 0.09 5-HT: 0.06 MEL: 0.009 DA: 0.34 OXT: 1.35 LOQ (pg mg^−1^) Nic: 0.15 Cot: 0.07 TES: 0.16 DHEA: 1.0 CRT: 0.29 5-HT: 0.18 MEL: 0.03 DA: 1.1 OXT: 4.4	Intra-day Nic: 2.4–7.2 Cot: 2.6–4.6 TES: 2.0–4.6 DHEA: 3.4–6.8 CRT: 2.2–2.9 5-HT: 4.7–5.5 MEL: 2.4–3.6 DA: 4.3–5.5 OXT: 3.6–5.5 Inter-day Nic: 4.3–9.8 Cot: 1.7–4.2 TES: 2.1–4.9 DHEA: 5.6–7.3 CRT: 3.3–5.7 5-HT: 5.4–6.5 MEL: 1.6–4.4 DA: 7.2–15.5 OXT: 4.5–10.5	Nic: 94.7–107.2 Cot: 91.5–108.2 TES: 90.7–100.5 DHEA: 95.2–101.1 CRT: 94.5–97.2 5-HT: 91.2–106.6 MEL: 84.0–95.9 DA: 81.1–93.9 OXT: 99.5–102.6	Non-smokers (7) Nic: 8.7–113 Cot: 0.0–32.9 TES: 0.0–6.2 DHEA: 1.0–7.5 CRT: 1.6–13.5 5-HT: 0.0–0.6 MEL: 0.00–0.09 DA: 0.0–6.4 OXT: 0.0–37.7 Smokers (3) Nic: 322–969 Cot: 254–726 TES: 3.7–7.7 DHEA: 2.3–5.3 CRT: 10.3–30.0 5-HT: 0.5–1.6 MEL: 0.03–0.07 DA: 4.5–18.9 OXT: 5.0–23.0	This study

^1^ Nic: nicotine; Cot: cotinine; TES: testosterone; DHEA: dehydroepiandrosterone; CRT: cortisol; 5-HT: serotonin; MEL: melatonin; DA: dopamine; OXT: oxytocin. ^2^ SIM: selected ion monitoring; MRM: multiple reaction monitoring; SRM: selected reaction monitoring; UPLC: ultra-performance liquid chromatography; APCI: atmospheric pressure chemical ionization. ^3^ LOD: limits of detection; LOQ: limits of quantitation; LLOQ: lower limits of quantitation. ^4^ Numbers in parentheses indicate the number of participants.

## 2. Results and Discussion

### 2.1. Optimization of IT-SPME and Desorption for Biomarkers

The IT-SPME system used in this study fully automates extraction, concentration, and desorption of target analytes in the capillary tube, followed by injection into the LC column via an autosampler. This configuration enables continuous analysis of multiple samples without the use of organic solvents [79]. The capillary tube was conditioned immediately before each sample analysis by washing with methanol and water to eliminate residue from previous samples. The air gap incorporated in the conditioning program was necessary to prevent mixing of the mobile phase and sample solution and to avoid desorption of analytes extracted onto the capillary coating during the extraction step. When a 40 µL sample was used, a 0.32 mm ID, 60 cm long capillary tube (internal volume: 48 µL) effectively prevented contamination of the sample loop and metering pump. To optimize IT-SPME conditions, capillary coatings, the number of draw/eject cycles and flow rate, and sample pH were investigated using a standard mixture containing 5.0 ng mL^−1^ Nic, 2.5 ng mL^−1^ Cot, 2.5 ng mL^−1^ TES, 10 ng mL^−1^ DHEA, 5.0 ng mL^−1^ CRT, 2.5 ng mL^−1^ 5-HT, 0.25 ng mL^−1^ MEL, 10 ng mL^−1^ DA, and 100 ng mL^−1^ OXT. The extraction efficiency for each biomarker was evaluated by calculating the enrichment factor, defined as the ratio of the peak height obtained by IT-SPME to that obtained by direct injection (10 μL) of the standard mixture.

**Figure 2:**
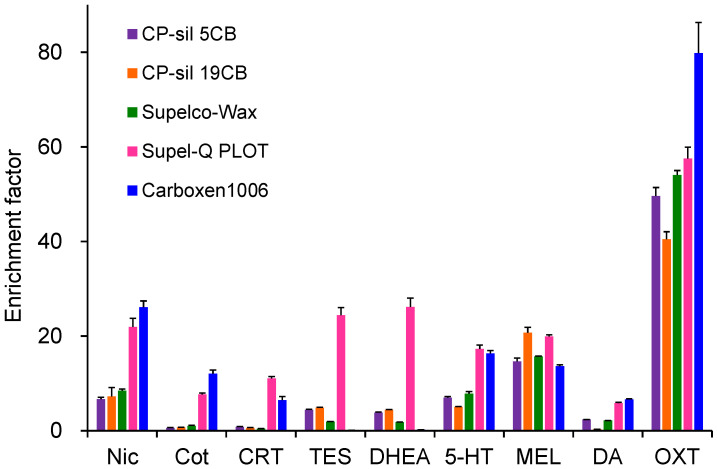
Effects of capillary coatings on IT-SPME of tobacco smoke exposure biomarkers and stress- and relaxation-related biomarkers. Extraction was performed by 25 draw/eject cycles of 40 μL of standard solution.

Extraction efficiencies were evaluated using five commercially available GC capillary columns (Figure 2). MEL and OXT showed high extraction efficiency on all capillaries, with OXT exhibiting the highest enrichment on Carboxen 1006, a porous adsorbent. In contrast, Nic, Cot, CRT, 5-HT, and DA showed low enrichment on CP-Sil 5CB, CP-Sil 19 CB, and CP-Wax 52CB, which were coated with thin-film liquid-phase adsorbents. Supel-Q PLOT and Carboxen 1006, both coated with thick-film porous adsorbents having large adsorption surface areas, showed relatively high enrichment for these compounds. However, Carboxen 1006 showed negligible enrichment for TES and DHEA and exhibited instability due to partial peeling of the coating from the capillary inner wall. Therefore, Supel-Q PLOT was selected as the optimal extraction device. The choice of capillary ID and length depends on the sample solution loading volume. Larger IDs reduce the contact area between the sample solution and the coating, decreasing extraction efficiency, whereas excessive length increases band broadening and extraction time. A capillary tube with 60 cm long with 0.32 mm ID was therefore optimal for loading a 40 µL sample [39].

**Figure 3:**
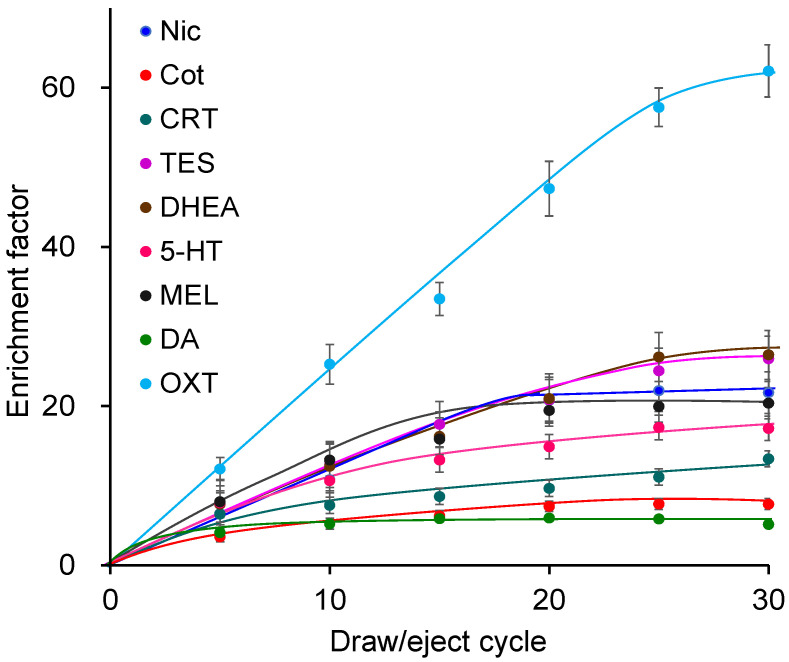
Effects of draw/eject cycles on IT-SPME of tobacco smoke exposure biomarkers and stress- and relaxation-related biomarkers. Extraction was performed with Supel-Q PLOT capillary by draw/eject cycles of 40 μL of standard solution.

Using the Supel-Q PLOT capillary, the effects of the draw/eject cycle number (5, 10, 15, 20, 25, 30) were investigated. Increasing the number of cycles enhanced extraction and enrichment (Figure 3) but also prolonged extraction time; thus, 25 cycles were selected. The draw/eject flow rate was investigated at 0.1, 0.15, 0.2, and 0.25 mL min^−1^. Although slightly higher enrichment was observed at slower flow rates, the differences were not significant. Excessively slow flow rates increased the extraction time, whereas faster rates promoted bubble formation; therefore, 0.2 mL min^−1^ was selected. Regarding sample pH, extraction was most efficient under weakly acidic conditions [39]. Among buffers tested at pH 3–8, acetic acid buffer at pH 4 showed the highest extraction efficiency. The biomarkers extracted into the capillary were almost completely desorbed by the mobile phase and directly introduced into the LC column, with no carryover observed. Under the optimized IT-SPME conditions, enrichment factors of 6–58 were achieved, with OXT showing the highest enrichment.

### 2.2. LC–MS/MS Analysis of Biomarkers

**Figure 4:**
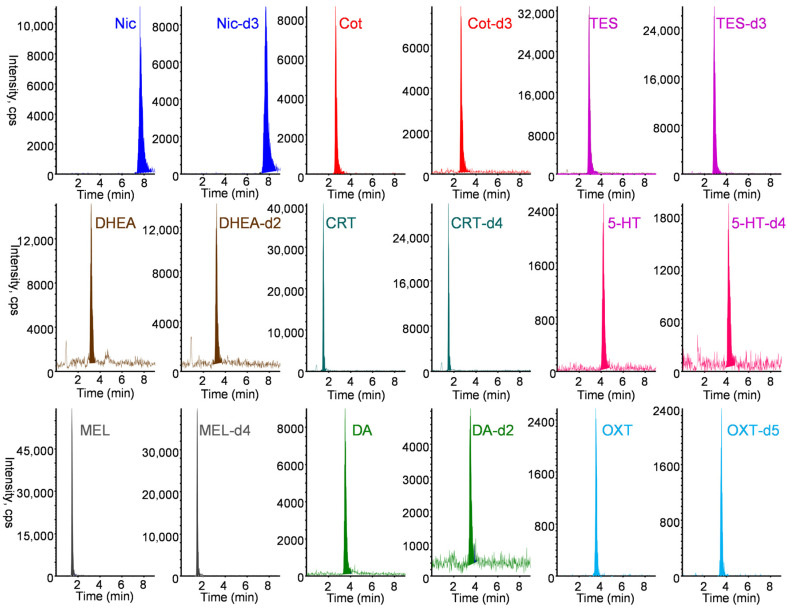
MRM chromatograms of a standard solution containing 10 ng mL^−1^ Nic, 5 ng mL^−1^ Cot, 10 ng mL^−1^ TES, 20 ng mL^−1^ DHEA, 10 ng mL^−1^ CRT, 10 ng mL^−1^ 5-HT, 0.5 ng mL^−1^ MEL, 20 ng mL^−1^ DA, and 200 ng mL^−1^ OXT and their stable isotope-labeled internal standard. IT-SPME LC–MS/MS conditions are described in Materials and Methods section.

All nine biomarkers and their stable isotope-labeled IS exhibited good sensitivity in positive ESI mode. The protonated molecular ion [M+H]+ and the most intense product ion generated from [M+H]+ were selected as the precursor (Q1) and product (Q3) ions, respectively. Chromatographic conditions were optimized to achieve rapid separation while minimizing matrix effects and maintaining good peak shape. Among the tested LC columns, the Discovery HS F5 column (50 mm × 2.1 mm) provided optimal separation and peak shape. As shown in Figure 4, the nine biomarkers and their stable isotope-labeled compounds were selectively analyzed in MRM mode within 7 min using a mobile phase of 0.1% formic acid/acetonitrile (60/40, *v*/*v*) at a flow rate of 0.2 mL min^−1^. Under the optimized online IT-SPME LC–MS/MS conditions, the total analysis time per sample was approximately 25 min, allowing automated analysis of approximately 57 samples per day and resulting in substantial labor savings, respectively. Chromatographic conditions were optimized to achieve rapid separation while minimizing matrix effects and maintaining good peak shape. Among the tested LC columns, the Discovery HS F5 column (50 mm × 2.1 mm) provided optimal separation and peak shape. As shown in Figure 4, the nine biomarkers and their stable isotope-labeled compounds were selectively analyzed in MRM mode within 7 min using a mobile phase of 0.1% formic acid/acetonitrile (60/40, *v*/*v*) at a flow rate of 0.2 mL min^−1^. Under the optimized online IT-SPME LC–MS/MS conditions, the total analysis time per sample was approximately 25 min, allowing automated analysis of approximately 57 samples per day and resulting in substantial labor savings.

### 2.3. Validation of the Developed Analytical Method

The performance of the developed IT-SPME LC–MS/MS method was evaluated in terms of linearity, sensitivity, and intra-day and inter-day precision (Table 2). Calibration curves were linear over the concentration range of 0.005–100 ng mL^−1^, with correlation coefficients of 0.9972 or higher (*n* = 18). Limits of detection ranged from 0.09 to 13.5 pg mL^−1^ at an S/N of 3, representing 6–58-fold higher sensitivity than the direct injection method (10 μL injection). Intra- and inter-day precision values (RSD, %) across low to high concentration ranges were 2.0–7.2% and 1.6–15.5%, respectively, demonstrating acceptable quantitative analysis precision.

### 2.4. Application to Hair Sample Analysis

**Figure 5:**
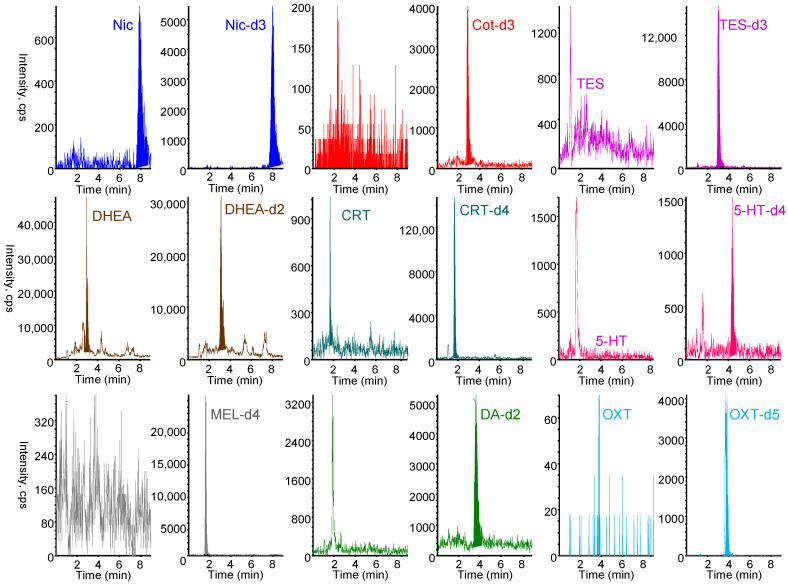
MRM chromatograms obtained from 5 mg hair. IT-SPME LC–MS/MS conditions are described in Materials and Methods section.

Hair is a suitable biomaterial for assessing chronic exposure and biological levels, as tobacco smoke exposure biomarkers and stress- and relaxation-related biomarkers are retained in hair for extended periods [7,8,43,44,45,46,47,48,49,50,51,52,53]. However, their concentrations in hair are substantially lower than in other biological matrices such as urine, blood, or saliva, necessitating efficient extraction, concentration, and highly sensitive analytical techniques. Table 1 summarizes previously reported analytical methods for biomarkers in hair samples. Although no method has been reported for the simultaneous analysis of tobacco smoke exposure biomarkers and stress- and relaxation-related biomarkers in hair, we previously developed a method for simultaneous analysis of seven biomarkers (Nic, Cot, CRT, 5-HT, MEL, DA, OXT) in saliva [39]. In the present study, this approach was expanded to include TES and DHEA, resulting in a method for nine biomarkers in hair samples. Hair samples were extracted with methanol and ultrafiltered using Amicon Ultra^®^ devices to remove high molecular weight proteins that could interfere with the extraction of low molecular weight biomarkers into the capillary tube. During methanol extraction of biomarkers from hair, minimal degradation was observed for each biomarker (Table S1). Biomarkers in the ultrafiltrate remained stable when stored at −20 °C until analysis. Furthermore, IT-SPME enabled the selective adsorption and concentration of biomarkers onto the capillary from hair extracts containing various matrix components. When adding stable isotope-labeled biomarkers (IS mixture solution shown in Section 3.1) to hair methanol extracts, the recovery rates obtained from their peak counts ranged from 79 to 91% (Table S1). Therefore, matrix effects in LC–MS/MS analysis were minimized by selective IT-SPME and corrected using stable isotope-labeled IS compounds, which exhibited behavior similar to that of the target biomarkers. As shown in Figure 5, biomarkers in hair samples from non-smokers were selectively detected, although background noise was noticeable in samples with low biomarker content. Quantification limits (S/N = 10) ranged from 0.03 to 4.4 pg mg^−1^ hair (Table 2), exceeding previously reported sensitivities [50,59,60,61,62,63]. The recovery of biomarkers spiked into hair samples ranged from 84.0 to 108.8%, with RSDs of 0.5–4.8% (Table 3), demonstrating accurate and quantitative performance.

**Figure 6:**
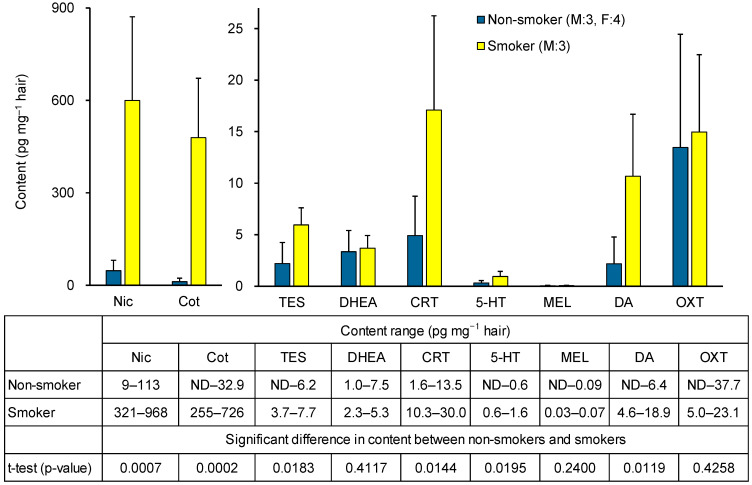
Hair biomarker contents in non-smokers and smokers, and significant differences between the two groups.

To assess the applicability of the method, a pilot study measured biomarker concentrations in hair from non-smokers (three males, four females) and smokers (three males). As shown in Figure 6, Nic and Cot levels were significantly higher in smokers. Among stress-related biomarkers, TES and CRT levels were significantly elevated in smokers, whereas no significant difference was observed for DHEA. The higher TES levels in smokers may reflect the fact that all smokers were male, whereas the non-smoker group included females. In fact, no significant differences in TES concentrations were observed between non-smokers and smokers in men alone. Among relaxation-related biomarkers, DA levels were significantly higher in smokers, while 5-HT and MEL levels were low, and OXT showed no significant difference. Reports on the content of 5-HT, MEL, and OXT in hair are scarce, with MEL being below the pg level [37] (Table 1). As shown in Table S2, a strong positive correlation (r = 0.9904) was observed between Nic and Cot levels in hair, and a positive correlation was also observed between Nic and CRT or DA levels. In contrast, no clear correlation was observed between hair levels of 5-HT, MEL, or OXT and Nic, Cot, or other stress-related biomarkers (Table S2). These results suggest that hair CRT and DA levels may be useful markers for examining the association with chronic tobacco smoke exposure, but that there is little correlation between tobacco smoke exposure and hair levels of 5-HT, MEL, or OXT. In smokers, Nic levels are elevated daily due to smoking, leading to repeated increases in CRT and DA induced by nicotine, which accumulate in hair. Previous studies have reported correlations between CRT levels in hair and saliva [44]. Our prior saliva analysis [42] showed that in non-smokers, smoking one cigarette increases CRT levels, and DA levels rise transiently but then decrease. These findings support the results of this study and suggest that although smoking may provide transient relaxation through short-term DA elevation, chronic tobacco smoke exposure ultimately functions as a stressor by increasing CRT levels. However, as both were pilot investigations, further studies with larger sample sizes are required to confirm these results.

## 3. Materials and Methods

### 3.1. Reagents and Materials

The targeted biomarkers for tobacco smoke exposure and stress/relaxation are shown in Figure 6. Nic, Cot, CRT, 5-HT, MEL and DA were purchased from Sigma-Aldrich Japan (Tokyo, Japan). TES was purchased from Nacalai Tesque (Kyoto, Japan), DHEA from Tokyo Chemical Industry (Tokyo, Japan), and OXT from Peptide Research Institute (Osaka, Japan). Nic-d3 (isotopic purity (IP) 98.4%), Cot-d3 (IP 99.9%), CRT-d4 (IP 99%), MEL-d4 (IP 98.7%), DA-d2 hydrochloride (IP 99.5%), and OXT-d5 trifluoroacetate (IP 98.9%) were purchased from Toronto Research Chemicals (Toronto, ON, Canada). TES-d3 (IP > 98%) was purchased from Sigma-Aldrich Japan, DHEA-d2 hydrochloride (IP 97%) from CDN Isotope Inc. (Quebec, QC, Canada), and 5-HT-d4 (IP > 99%) from Cayman Chemical (Ann Arbor, MI, USA) and used as internal standards (IS). Each standard and IS compound, except OXT and OXT-d5, was dissolved in LC–MS grade methanol (Kanto Chemical, Tokyo, Japan) at 0.5 mg mL^−1^. OXT and OXT-d5 were dissolved in LC–MS grade distilled water (Kanto Chemical) at 0.1 mg mL^−1^. These stock solutions were stored in tightly sealed brown screw-cap vials at −20 °C. Prior to use, they were diluted with distilled water to the required concentration to prepare working standard mixtures. The IS mixture was prepared by diluting 10 ng mL^−1^ Nic-d3, 5 ng mL^−1^ Cot-d_3_, 10 ng mL^−1^ TES-d3, 20 ng mL^−1^ DHEA-d2, 10 ng mL^−1^ CRT-d4, 10 ng mL^−1^ 5-HT-d4, 0.5 ng mL^−1^ MEL-d4, 20 ng mL^−1^ DA-d2, and 200 ng mL^−1^ OXT-d5 with LC–MS grade distilled water. These solutions were tightly sealed and stored at 4 °C until use. HPLC grade formic acid (Nacalai Tesque), LC–MS grade distilled water, and LC–MS grade acetonitrile (Kanto Chemical) were used as mobile phase solvents.

A commercially available GC capillary column (60 cm × 0.32 mm ID) was used as the extraction device for IT-SPME. CP-Sil 5CB (100% polydimethylsiloxane, film thickness (FT) 5 μm), CP-Sil 19CB (14% cyanopropyl phenyl methylsilicone, FT 1.2 μm), and CP-Wax 52CB (polyethylene glycol, FT 1.2 μm) were purchased from Varian Inc. (Lake Forest, CA, USA). Supel-Q PLOT (divinylbenzene polymer, FT 17 μm) and Carboxen 1006 PLOT (Carboxen molecular adsorbent, FT 15 μm) were purchased from Supelco (Bellefonte, PA, USA).

### 3.2. LC–MS/MS Instrument and Analytical Conditions

LC–MS/MS analysis was performed using an Agilent 1100 Series LC system (Agilent Technologies, Böblingen, Germany) coupled to an API 4000 triple quadrupole mass spectrometer (AB SCIEX, Foster City, CA, USA). Nitrogen generated from compressed air by a Kaken N_2_ generator (System Instruments Co., Ltd., Tokyo, Japan) was used as the nebulizer and drying gas. Data acquisition and processing were conducted using Analyst Software 1.6.2 (AB SCIEX). Chromatographic separation was achieved on a Discovery HS F5-3 column (50 mm × 2.1 mm, 3 μm particle size) maintained at 40 °C, using a mobile phase of 0.1% formic acid/acetonitrile (60/40, *v*/*v*) at a flow rate of 0.2 mL min^−1^ for 7 min. Electrospray ionization (ESI)-MS/MS was performed for the nine biomarkers and their stable isotope-labeled compounds under the following conditions: ion spray voltage 5500 V, source temperature 600 °C, GS1 50 L min^−1^, GS2 80 L min^−1^, curtain gas 30 L min^−1^, collision gas 4 L min^−1^, and a dwell time 55 ms. Optimized MS/MS parameters, including multiple reaction monitoring (MRM) transitions in positive ion mode, are summarized in Table 4. Quantification was based on MRM of the protonated precursor ion [M+H]^+^ (Q1) and the corresponding product ion (Q3).

### 3.3. IT-SPME Procedure and Online Coupled Analysis with LC–MS/MS

**Figure 7:**
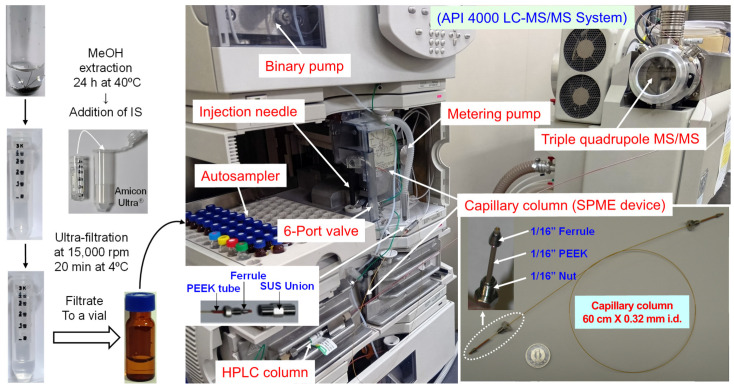
Outline of hair sample preparation and online IT-SPME LC–MS/MS system.

Figure 7 illustrates an online IT-SPME LC–MS/MS system incorporating a capillary column as the extraction device into an HPLC autosampler. Both ends of a GC Supel-Q PLOT capillary column (60 cm long, 0.32 mm inner diameter (ID), 48 μL internal volume) were threaded into 1/16-inch polyether ether ketone tubing sleeves (2.5 cm long, 330 μm ID) and connected between the autosampler injection loop and injection needle using standard 1/16-inch stainless steel nuts, ferrules, and union connectors. The injection loop was retained to prevent contamination of the metering pump by the sample. The autosampler software (Analyst 1.6.2, AB SCIEX) was programmed to control SPME extraction, desorption, and injection within the tubing. The extraction and concentration procedure is shown in Table 5.

A 2 mL brown screw-cap autosampler vial containing 0.5 mL of sample solution and fitted with a silicone/polytetrafluoroethylene septum was placed in the autosampler sample tray. Four additional autosampler vials were also placed in the tray: two containing 1.5 mL methanol (A, D), one containing 1.5 mL distilled water (C), and one blank vial (B). Before sample extraction, the 6-port valve was set to the LOAD position, and the capillary column was washed and conditioned at a flow rate of 0.2 mL min^−1^ as follows: (1) drawing/ejecting 40 μL methanol (A) for 2 cycles; (2) drawing 50 μL air from the blank vial (B) and (C) drawing/ejecting 40 μL distilled water (C) for 2 cycles. Subsequently, with the 6-port valve remaining in the LOAD position, extraction was performed by drawing/ejecting 40 μL of sample 25 times at a flow rate of 0.2 mL min^−1^ to extract the target biomarkers and IS compounds onto the capillary coating. After extraction, the injection needle tip was washed once by drawing/ejecting 2 μL methanol (D) from another autosampler vial. Finally, the compounds extracted onto the capillary column were desorbed from the capillary coating by the mobile phase flow when the 6-port valve was switched to the INJECT position, and were subsequently transferred to the LC–MS/MS system. All IT-SPME and LC–MS/MS steps, including capillary column conditioning, extraction, desorption, injection, chromatographic separation and detection, were fully automated by the autosampler software.

### 3.4. Method Validation Study

The developed method was evaluated in terms of linearity, limit of detection, limit of quantitation, and precision. Linearity was evaluated by triplicate analyses of six concentration levels for each analyte: Nic and CRT (0.1, 0.2, 0.5, 1.0, 2.0, 5.0 ng mL^−1^), Cot, TES, and 5-HT (0.05, 0.1, 0.25, 0.5, 1.0, 2.5 ng mL^−1^), DHEA and DA (0.2, 0.4, 1.0, 2.0, 4.0, 10 ng mL^−1^), MEL (0.005, 0.01, 0.025, 0.05, 0.1, 0.25 ng mL^−1^), OXT (2, 4, 10, 20, 40, 100 ng mL^−1^). For each vial, 0.05 mL IS mixture and 0.05 mL 0.2 M acetate buffer (pH 4) were added, and the total volume was adjusted to 0.5 mL with distilled water. The vials were then placed in the autosampler and analyzed. Calibration curves were constructed using the peak height ratios of each analyte to its corresponding IS at each concentration level. Method sensitivity was assessed by determining the limits of detection and quantitation, defined as signal-to-noise ratios (S/N) of 3 and 10, respectively. Intra-day precision was evaluated by six independent analyses on the same day, and inter-day precision on six analyses over six days. Precision was expressed as relative standard deviation (RSD, %) and assessed at three concentration levels (low, medium, high) for each analyte.

### 3.5. Collection, Preparation, and Analysis of Hair Samples

Informed consent was obtained from all participants, and the study was approved by the Shujitsu University Ethics Committee. Hair samples were collected from six healthy males (3 smokers, 3 non-smokers) and four females (non-smokers) aged 22–30 years, and separately stored in sealed plastic bags. The collected hair samples were sonicated twice for 3 min in dichloromethane to remove external contaminants, then spread on filter paper and dried at room temperature. The dried hair was cut into 2–3 mm segments using scissors. Fifteen milligrams of each sample were placed into 9 mL screw-cap vials, 0.6 mL methanol was added, and the mixture was shaken and extracted at 40 °C for 24 h. After adding 0.15 mL of IS mixture to the methanol extract, the mixture was ultrafiltered using an Amicon Ultra^®^ 0.5 mL 3K regenerated cellulose centrifugal filter device (molecular weight cut-off 3000; Millipore, Cork, Ireland) at 15,000 rpm for 20 min. Subsequently, 0.2 mL of the filtrate (corresponding to 5 mg hair) was transferred to a 2.0 mL brown autosampler vial fitted with a septum and evaporated to dryness under a nitrogen stream at room temperature. The residue was reconstituted with 0.05 mL 0.2 M acetate buffer (pH 4) and distilled water to a final volume of 0.5 mL, and then subjected to IT-SPME LC–MS/MS analysis. The concentrations of target biomarkers in hair were quantified using calibration curves and expressed as content per milligram of hair.

### 3.6. Statistical Analysis

Statistical analysis of all measurement data was performed using Microsoft Excel 2019. Pearson correlation coefficients (r) were calculated to evaluate the linear association between tobacco smoke exposure marker concentrations and stress- and relaxation-related biomarker concentrations. An r value ≥ 0.8 was considered a strong positive correlation, ≤−0.8 a strong negative correlation, and a range of −0.2 to 0.2 was considered no correlation. Additionally, *p*-values were calculated using Student’s *t*-tests to evaluate significant differences in the mean concentrations of each biomarker between non-smokers and smokers. A two-tailed *p*-value < 0.05 was considered statistically significant.

## 4. Conclusions

In this study, we developed a novel analytical method for the simultaneous measurement of hair biomarkers related to tobacco smoke exposure and stress/relaxation in order to elucidate the relationship between long-term tobacco smoke exposure and chronic stress. The proposed method achieves full automation of sample extraction and concentration, chromatographic separation, detection, and data analysis through online coupling of IT-SPME with LC–MS/MS. This configuration enables unattended overnight operation, reduces solvent consumption and labor costs, and improves analytical efficiency. The method uniquely allows non-invasive, highly sensitive, and precise analysis of nine biomarkers at the picogram level using only ultrafiltration of a small volume methanol hair extract. Consequently, tobacco smoke exposure and stress-related biomarker levels can be objectively assessed in a single analysis using the same sample. This is the first study to simultaneously analyze tobacco smoke exposure biomarkers and stress and relaxation biomarkers in hair. A pilot analysis of hair samples from smokers and non-smokers reflected the association with chronic tobacco smoke exposure, which acts as a stressor. With analysis of larger sample sets in future studies, the biomarker analysis method developed here has the potential to become a valuable tool not only for investigating the relationship between tobacco smoke exposure and stress, but also for the early diagnosis and prevention of stress-related diseases.

## Figures and Tables

**Table 2:** Validation of IT-SPME/LC–MS/MS method for biomarkers.

Analyte	Linearity (ng mL^−1^)		LOD ^2^ (pg mL^−1^)	LOQ ^3^ (pg mg^−1^)	Precision (RSD%), (*n* = 6)					
					Intra-Day			Inter-Day		
	Range	CC ^1^			Low	Medium	High	Low	Medium	High
Nic	0.1–5.0	0.9999	0.46	0.15	7.2	3.9	2.4	8.8	9.8	4.3
Cot	0.05–2.5	0.9996	0.21	0.07	3.0	2.6	4.6	4.2	2.6	1.7
TES	0.05–2.5	1.0000	0.48	0.16	4.0	2.0	4.6	4.4	4.9	2.1
DHEA	0.2–10	0.9996	3.1	1.0	3.4	5.3	6.8	6.9	7.3	5.6
CRT	0.1–5.0	1.0000	0.89	0.29	2.9	2.2	2.8	5.7	3.3	3.9
5-HT	0.05–2.5	0.9997	0.56	0.18	5.3	4.7	5.5	6.1	6.5	5.4
MEL	0.005–0.25	1.0000	0.09	0.03	2.4	3.6	2.9	4.4	3.8	1.6
DA	0.2–10	0.9972	3.4	1.1	5.5	4.3	5.5	15.5	7.2	11.8
OXT	2–100	0.9994	13.5	4.4	3.9	3.6	5.6	10.5	6.7	4.5

^1^ Correlation coefficient (*n* = 18). ^2^ Limits of detection: pg mL^−1^ standard solution (S/N, 3). ^3^ Limits of quantification: pg mg^−1^ hair sample (S/N, 10).

**Table 3:** Recoveries of biomarkers spiked into hair samples.

Analyte	Spiked (ng mg^−1^ Hair)	Recovery ± SD (%), (*n* = 3)
Nic	0.02	94.7 ± 2.3
	0.10	104.9 ± 0.7
	0.50	107.2 ± 0.9
Cot	0.01	91.5 ± 3.5
	0.05	94.7 ± 0.7
	0.25	108.8 ± 0.5
TES	0.01	100.5 ± 1.5
	0.05	93.2 ± 4.2
	0.25	90.7 ± 1.1
DHEA	0.04	95.2 ± 2.2
	0.20	96.2 ± 1.4
	1.00	101.1 ± 2.7
CRT	0.02	97.2 ± 1.4
	0.10	96.1 ± 4.3
	0.50	94.5 ± 1.1
5-HT	0.01	91.2 ± 2.1
	0.05	106.6 ± 1.6
	0.25	99.6 ± 2.6
MEL	0.001	95.9 ± 0.5
	0.005	86.0 ± 0.7
	0.025	84.0 ± 4.8
DA	0.04	89.9 ± 2.9
	0.20	93.9 ± 3.8
	1.00	81.1 ± 1.1
OXT	0.4	101.9 ± 4.1
	2.0	99.5 ± 1.0
	10.0	102.6 ± 2.7

**Table 4:** MRM transitions and setting parameters for target biomarkers and their stable isotope-labeled compounds.

Compound	Mass Transition (*m*/*z*)	DP ^1^ (V)	EP ^2^ (V)	CE ^3^ (V)	CXP ^4^ (V)
Nicotine (Nic)	163.1 → 132.1	70	10	20	10
Nic-d3	166.1 → 132.1	70	10	20	10
Cotinine (Cot)	177.1 → 80.2	75	10	30	15
Cot-d3	180.1 → 80.2	75	10	30	15
Testosterone (TES)	289.0 → 109.0	70	10	35	10
TES-d3	292.0 → 109.4	70	10	35	10
Dehydroepiandrosterone (DHEA)	289.4→ 271.4	40	10	13	10
DHEA-d2	291.4 →273.4	40	10	13	10
Cortisol (CRT)	363.0 → 120.9	70	10	30	10
CRT-d4	367.1 → 121.4	70	10	30	10
Serotonin (5-HT)	177.2 → 160.2	25	5	15	3
5-HT-d4	181.2 → 164.3	25	5	15	3
Melatonin (MEL)	233.1 → 174.1	20	9	20	12
MEL-d4	237.1 → 178.1	20	9	20	12
Dopamine (DA)	154.2 → 91.1	50	4	30	8
DA-d2	156.2 → 93.1	50	4	30	8
Oxytocin (OXT)	1008.3 → 724.5	60	9	40	12
OXT-d5	1013.3 → 724.5	60	9	40	12

^1^ Declustering potential. ^2^ Entrance potential. ^3^ Collision energy. ^4^ Collision cell exit potential.

**Table 5:** Program for the IT-SPME process.

Sequence	Event	Switching Valve	Vial	Draw/Eject		
				Cycle ^1^	Volume (μL)	Speed (μL min^−1^)
1	Conditioning of the capillary	Load	MeOH (A)	D/E (2)	40	200
2	Drawing of air into the capillary	Load	Empty (B)	D (1)	50	200
3	Conditioning of the capillary	Load	Water (C)	D/E (2)	40	200
4	Extraction of analytes into the capillary	Load	Sample	D/E (25)	40	200
5	Needle washing	Load	MeOH (D)	D/E (1)	2	200
6	Desorption of analytes from the capillary	Inject	−	−	−	−
7	HPLC separation of analytes and return to sequence 1	Load	−	−	−	−

^1^ D: draw, E: ejection.

## Data Availability

The data presented in this study are available on request from the corresponding author.

## Supplementary Materials

The following supporting information can be downloaded at https://www.mdpi.com/article/10.3390/molecules31050770/s1, Table S1: Stabilities of biomarkers in methanol solution and matrix effects in LC-MS/MS; Table S2: Correlation between concentrations of each marker for tobacco smoke exposure markers and stress- and relaxation-related biomarkers obtained from all subjects (*n* = 10).
