# Supplementary material for: Simultaneous Analysis of Biomarkers in Human Hair for Evaluating Chronic Tobacco Smoke Exposure and Stress/Relaxation Using Online In-Tube Solid-Phase Microextraction Coupled with Liquid Chromatography–Tandem Mass Spectrometry
Source: Molecules. 2026 Feb 25;31(5):770. doi: 10.3390/molecules31050770 (PMC12986007; doi:10.3390/molecules31050770)
Supplement: Supplementary file 1 [file molecules-31-00770-s001.zip › molecules-4155268-supplementary.pdf]

Supplementary data (molecules-4155268)

Simultaneous Analysis of Biomarkers in Human Hair for Evaluating Chronic Tobacco Smoke Exposure and Stress/Relaxation Using Online In-Tube Solid-Phase Microextraction Coupled with Liquid Chromatography–Tandem Mass Spectrometry

Hiroyuki Kataoka *, Akiko Tsuzaki, Sae Kitagawa, Kentaro Ehara

Table S1. Stabilities of biomarkers in methanol solution and matrix effects in LC-MS/MS.

Stabilities of biomarkers[1]			Matrix effects[2]		
Biomarker	Spiked ($ng\ mL^{-1}$)	Recovery ± SD (%), (n = 3)	Labeled biomarker	Spiked ($ng\ mg^{-1}$ hair)	Recovery ± SD (%), (n = 3)
Nic	1.0	99.5 ± 5.2	Nic-d3	0.10	82.8 ± 1.9
Cot	0.5	87.3 ± 4.0	Cot-d3	0.05	90.0 ± 3.4
TES	2.0	89.3 ± 2.5	TES-d3	0.20	83.1 ± 1.4
DHEA	1.0	97.8 ± 5.5	DHEA-d2	0.10	85.1 ± 1.2
CRT	0.5	101.9 ± 3.1	CRT-d4	0.05	90.8 ± 1.6
5-HT	0.05	97.2 ± 2.2	5-HT-d4	0.005	82.5 ± 1.5
MEL	2.0	104.6 ± 1.3	MEL-d4	0.20	87.8 ± 1.4
DA	20	88.6 ± 0.9	DA-d2	2.0	79.4 ± 1.9
OXT	100	95.8 ± 6.6	OXT-d5	10.0	81.3 ± 7.3

[1] Stabilities of biomarkers were expressed as the recovery rate after heating in methanol solution at 40°C for 24 h.
[2] Matrix effects were expressed as the recovery rate of the stable isotope-labeled biomarkers spiked to the methanol extract of hair.

Table S2. Correlation between concentrations of each marker for tobacco smoke exposure markers and stress- and relaxation-related biomarkers obtained from all subjects (*n*=10).

	Nic	Cot	TES	DHEA	CRT	5-HT	MEL	DA
Cot	0.9904							
TES	0.7303	0.7570						
DHEA	0.2107	0.1897	0.2131					
CRT	0.8952	0.8665	0.8377	0.3734				
5-HT	0.3848	0.4002	0.4113	0.1429	0.3535			
MEL	0.3984	0.3726	0.7805	0.2963	0.6260	0.3407		
DA	0.9277	0.8937	0.7574	0.4081	0.9293	0.3254	0.6211	
OXT	0.2628	0.2391	0.6352	0.4108	0.5874	–0.0549	0.7727	0.4706

Each data point represents the correlation between the respective biomarker concentrations as a Pearson correlation coefficient (r).
